# Highly Sensitive Voltammetric Method for Quinoline Yellow Determination on Renewable Amalgam Film Electrode

**DOI:** 10.3390/molecules28145475

**Published:** 2023-07-18

**Authors:** Anna Górska-Ratusznik, Dominika Różańska, Joanna Smajdor, Beata Paczosa-Bator, Robert Piech

**Affiliations:** 1 Sieć Badawcza Łukasiewicz—Krakowski Instytut Technologiczny, ul. Zakopiańska 73, 30-418 Cracow, Poland; 2 Faculty of Materials Science and Ceramics, AGH University of Science and Technology, al. Mickiewicza 30, 30-059 Cracow, Poland

**Keywords:** food coloring, quality control, quinoline yellow, voltammetry

## Abstract

A novel electrochemical method for the determination of quinoline yellow (QY) was developed using the renewable amalgam film electrode (Hg(Ag)FE). The sensors used can be characterized by good stability and long lifespan. Irreversible QY reduction peaks were recorded in 0.05 mol L^−1^ HCl with a potential of about −630 mV. The use of the Hg(Ag)FE electrode with a regulated working surface allowed the QY limit of detection to be as low as 0.48 nmol L^−1^. The obtained result is the lowest in comparison to other voltammetric methods described in the literature. The effects of parameters such as the size of the working electrode surface, influence of the pH value, accumulation time, and potential were investigated to provide precision and high sensitivity of the performed measurements. This new procedure was applied for the highly sensitive determination of quinoline yellow in different beverages, pre-workout supplements, and throat lozenges. The process of sample preparation was relatively simple. Calculated recoveries (96–107%) suggest that the method can be considered accurate.

## 1. Introduction

Many food additives are used in the modern food industry. Among them, there are coloring substances that have a positive effect on improving the appearance of food products, at the same time encouraging the consumer to buy. However, the use of synthetic organic dyes in food can negatively affect the human body. One of them, commonly used in food and beverages, is quinoline yellow (E 104) (QY). Scientific research has proven that the usage of QY may be connected to the possibility of causing a number of side effects, such as the possibility of allergic reactions, increased sensitivity to certain drug components (e.g., salicylates), increased hyperactivity in children, and even potential carcinogenic effects [1,2,3]. Therefore, the concentrations of QY and other colorants are regulated and tested in food products. 

In food technology, great emphasis is placed on controlling its quality and the concentrations of individual ingredients that affect the safety of consumption, to which food colorants and quinoline yellow itself belong. One of the fields of science that allows such activities to be monitored is analytical chemistry. Its essence is to strive to develop methods that will accurately and precisely determine the chemical composition of a given sample. In chemical analysis used for highly sensitive quinoline yellow determination, spectroscopic methods [4,5,6,7,8], chromatographic methods [9,10], or electrochemical methods [11,12,13,14,15,16,17,18,19] can be distinguished in particular. Among the electrochemical methods, voltammetry gained the greatest popularity due to its ease, high sensitivity, and short sample analysis time. 

In order to measure the low concentration of quinoline yellow and achieve a satisfactory detection limit, the renewable amalgam film electrode (Hg(Ag)FE) was used in this study. Among the variety of possible working electrode constructions, we can distinguish the solid ones, made from glassy carbon or noble metals, paste electrodes made from conductive powders mixed with mineral oil, or classical mercury electrodes. The renewable amalgam film electrode is a modification of the classic hanging mercury drop electrode (HMDE), which significantly reduces the amount of mercury waste while maintaining the high sensitivity and repeatability of the performed measurements. The Hg(Ag)FE electrode may be used both in organic and inorganic compound analysis in different matrices, maintaining the high sensitivity of the performed measurements. 

The proposed study presents a new, highly sensitive method for voltammetric determination of quinoline yellow. As a working electrode, a renewable amalgam film electrode was used. The proposed sensor provides a highly sensitive method as well as high precision and repeatability. The applicability of the developed voltammetric method was proven after the successful analyses of real samples, such as popular beverages, tablets, and powder samples. 

## 2. Results and Discussion

### 2.1. Quinoline Yellow Behavior on Hg(Ag)FE

**Figure 1:**
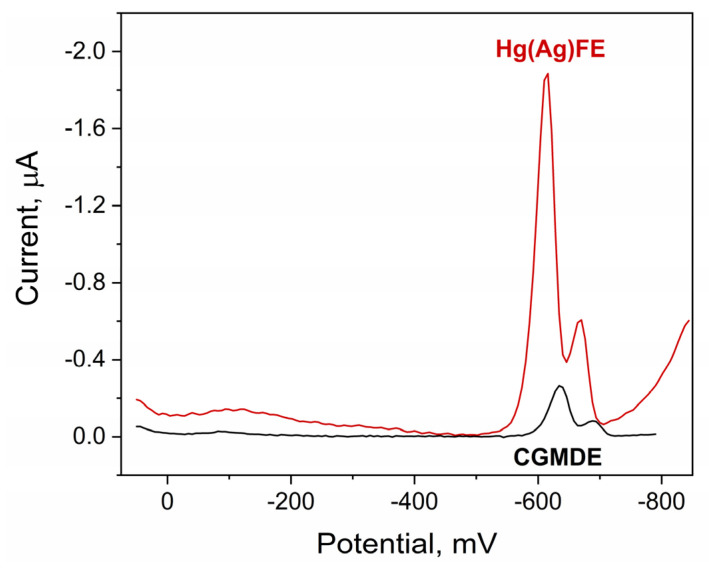
Comparison of 0.2 µmol L^−1^ quinoline yellow voltammograms in 0.05 mol L^−1^ HCl obtained for HMDE (black line) and Hg(Ag)FE (red line) electrode. The electrode areas were: 9.8 mm^2^ for the (Hg(Ag)FE) and 1.6 mm^2^ for the HMDE. Other instrumental parameters as in Section 3.4.

In order to provide the high sensitivity and repeatability of quinoline yellow measurements, the differential pulse voltammetric technique was applied. Two types of mercury electrodes—hanging mercury drop electrode (HMDE) and renewable amalgam film electrode (Hg(Ag)FE)—were tested to find the most suitable working electrode for QY determination. The signal of 0.2 µmol L^−1^ QY registered on both electrodes is presented in Figure 1. 

The quinoline yellow signals obtained on the Hg(Ag)FE and HMDE electrodes were compared. Current density and peak potential were equal to 0.17 µA mm^−2^, −628 mV, and 0.14 µA mm^−2^, −636 mV, respectively, for Hg(Ag)FE and HMDE. For further measurements, the Hg(Ag)FE electrode was chosen due to its parameters, higher current density, a significant reduction in toxic waste, ease of use, and long-term stability.

Mercury film electrode constructions allow one to change the size of the working surface; therefore, the measured QY signal values change with the change in this parameter. The measured signal of 0.2 µmol L^−1^ quinoline yellow grew linearly as the Hg(Ag)FE surface increased its size from 3.8 to 13.8 mm^2^, according to the equation:

I_p_ = 0.169 ± 0.09 [µA mm^−2^] − 0.026 ± 0.01 [µA], r = 0.993

**Figure 2:**
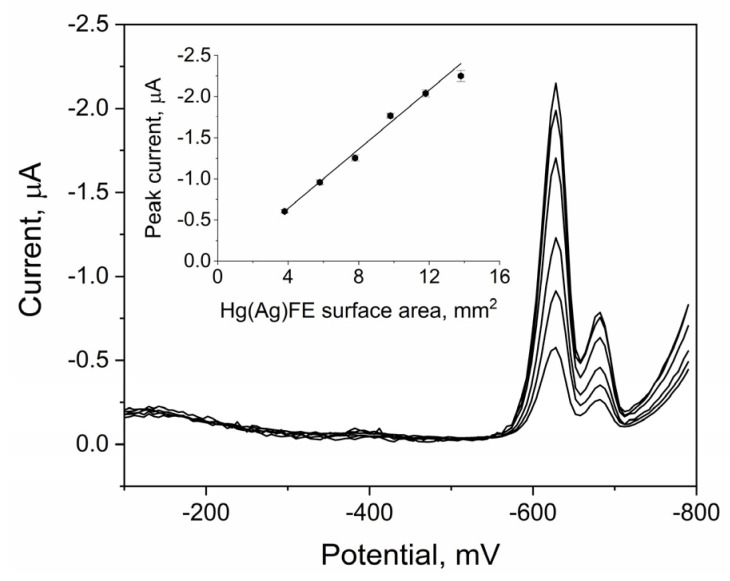
The signal of 0.2 µmol L^−1^ quinoline yellow measured in 0.05 mol L^−1^ HCl on Hg(Ag)FE electrode with different sizes of the working surface in a range from 3.8 mm^2^ to 13.8 mm^2^. Other instrumental parameters as in Section 3.4.

For further studies, an electrode surface area of 9.8 mm^2^ was chosen due to a good relationship of signal to background current and favorable peak parameters (Figure 2).

### 2.2. Cyclic Voltammetry

**Figure 3:**
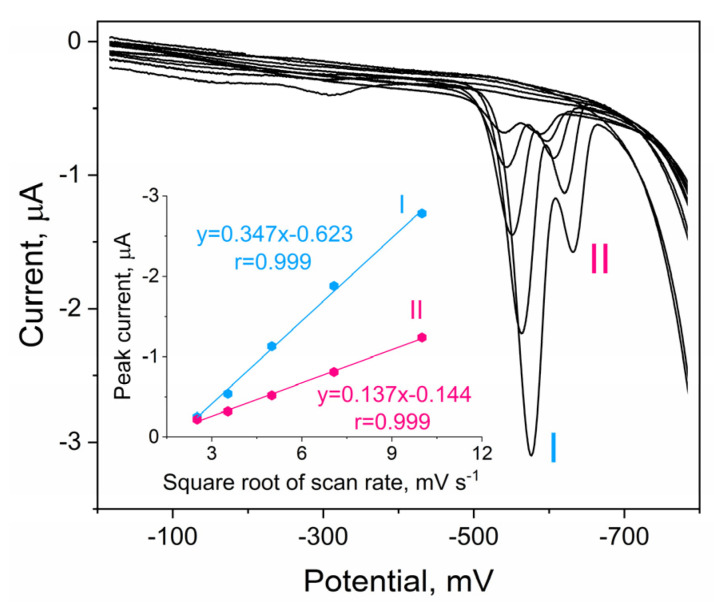
Cyclic voltammograms of 2.5 µmol L^−1^ quinoline yellow in 0.05 mL L^−1^ HCl registered on renewable mercury amalgam electrode for scan rate values of 6.3; 12.5; 25; 50; 100 mV s^−1^.

Quinoline yellow is a complex compound, and its electrochemical behavior is also complicated. In order to investigate the QY reduction mechanism on the renewable amalgam film electrode surface, linear sweep voltammetry (LSV) measurements were performed. The QY voltammograms registered with scan rate values in the range of 6.3 mV s^−1^ to 100 mV s^−1^ are presented in Figure 3. 

The absence of the QY oxidation peak in the anodic scan suggests that its reduction process is irreversible, whereas, in the cathodic waveform, two reduction peaks can be observed, which indicates a two-step reduction mechanism. To propose the possible QY reduction reaction mechanism, plots of the peak current versus the scan rate and the peak current versus the square root of the scan rate were investigated. For both registered peaks, the linear correlation between the peak current and the square root of the scan rate was obtained, which suggests that in both cases, the QY reduction reaction was controlled by diffusion. 

For both QY peaks, the number of electrons exchanged during the reduction process was calculated using the graphs plotted for the dependence of the peak current vs. the logarithm of the scan rate. The linear regression equations obtained were as follows:

(I) peak: E_k_ = 0.01429 ln*v* − 0.607 [V], r = 0.987

(II) peak: E_k_ = 0.01576 ln*v* − 0.665 [V], r = 0.997

The slope values obtained in the linear regression equations allow one to calculate the number of electrons exchanged in the unit reduction process. Assuming the α value as 0.5, the calculated number of electrons exchanged in each step of the QY reduction process equals two.

Moreover, for the I QY peak, a Tafel plot was crossed out in order to confirm the previous calculations. Potential versus peak current logarithm plot data were read from the increasing part of the quinoline yellow current–voltage curve registered on the Hg(Ag)FE electrode for the scan rate value of 50 mV s^−1^. The slope of the constructed regression line was equal to −0.05916, thus considering the equation:

*s* = (−2.3*RT*)/*nαF*

and assuming the α value for the irreversible reduction reaction as 0.5, it is possible to calculate the number of electrons exchanged in the reduction reaction. The number of calculated electrons was equal to 2, which is in good relation to previous results. For peak II, the construction of a Tafel plot was not possible because of QY peak overlapping and the lack of the peak II baseline.

**Figure 4:**
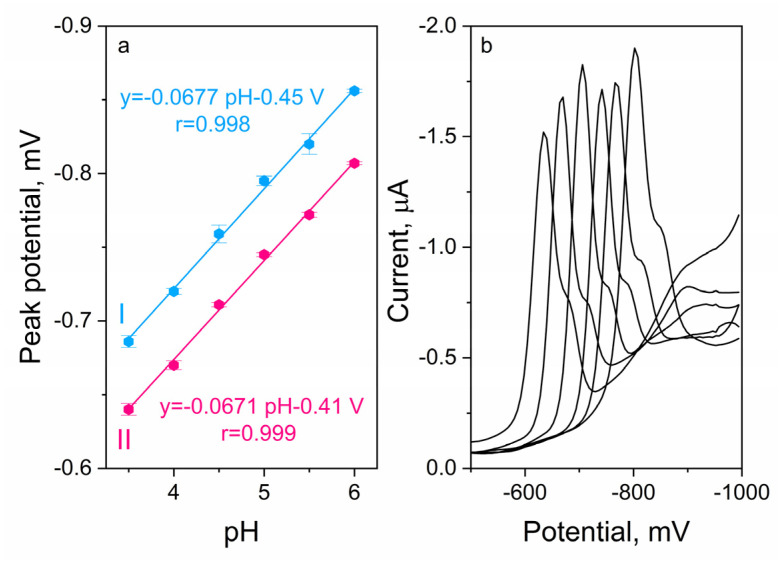
Dependence of the quinoline yellow peaks potential on pH measured in the pH range from 3.5 to 6.0 for 1 × 10^−7^ mol L^−1^ QY in 0.05 mol L^−1^ acetate buffer (**a**) and corresponding voltammograms (**b**). Other instrumental parameters as in Section 3.4.

To obtain information on the number of protons exchanged in the electrochemical reduction of quinoline yellow, the dependence between the pH value of the QY peaks potentials and the supporting electrolyte pH value was investigated (Figure 4).

DPV voltammograms of 1 × 10^−7^ mol L^−1^ QY were recorded in the acetate buffer electrolytes in the range of 3.5 to 6.0. The cathodic peak current values were slightly dependent on the pH values, e.g., for the pH value of 3.5, the I QY peak current was equal to 1.44 µA (minimum QY peak current value), whereas, for the pH value of 4.5, the QY peak current was equal to 1.75 µA (maximum QY peak value). For the II QY peak, there was no significant change in the current value. The QY peak potential was shifted toward more negative values with an increase in the pH value. For both QY peaks, the equations obtained for dependence between pH values and peak potential were linear in the form of:

(I) peak: E_p_ = 0.0667 pH − 0.45 V

(II) peak: E_p_ = 0.0671 pH − 0.41 V

**Scheme 1:**
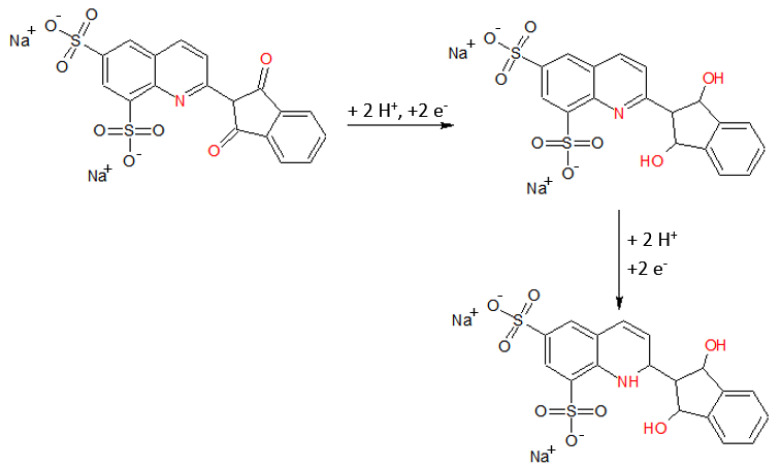
Quinoline Yellow proposed reduction reaction.

The slope parameter of the obtained equations was equal to 0.0677 (I peak) and 0.0671 (II peak), which was close to the theoretical value of 0.059. Therefore, it is possible to say that the number of protons exchanged in the QY reduction reaction equals the number of electrons. The possible two-step reduction process of quinoline yellow is presented in Scheme 1.

### 2.3. DPV Parameters Optimization

The differential pulse voltammetry (DPV) technique was chosen for quantitative measurements of QY, and the I peak was considered an analytical signal. In order to improve the sensitivity of the method and to establish the best conditions for the measurements, the parameters of the DPV technique were optimized. All experiments were conducted in 0.05 mol L^−1^ hydrochloric acid as a supporting electrolyte. The following parameters were optimized: waiting and sampling time (*t_w_* and *t_s_*) in the range 10–100 ms, step potential *E_s_* (1–6 mV), and pulse amplitude Δ*E* (5–100 mV, both positive and negative mode). According to the obtained results, optimal conditions for QY determination were as follows: t_p_ = *t_w_* = 10 ms, *E_s_* = 6 mV, Δ*E* = 50 mV; therefore, such values were used in further experiments.

### 2.4. Supporting Electrolyte Optimization

#### 2.4.1. Type of Supporting Electrolyte

The choice of the supporting electrolyte is an important part of developing new voltammetric methods; therefore, the appropriate experiments were conducted. During each measurement, preconcentration potential and time were equal to 50 mV and 20 s, respectively, and QY concentration was equal to 0.2 µmol L^−1^. The following electrolytes were tested: 0.05 mol L^−1^ HCl, 0.05 mol L^−1^ KCl, 0.05 mol L^−1^ KH_2_PO_4_, 0.05 mol L^−1^ acetate buffer (pH 3.8), 0.05 mol L^−1^ ammonia buffer (pH 8.2), 0.05 mol L^−1^ borate buffer (pH 9.1). In the potential range between 50 mV to −850 mV, the peak derived from QY was registered in HCl and acetate buffer. In the remaining electrolytes, the QY peak was not observed. Due to the fact that in 0.05 mol L^−1^ HCl, the QY peak was well-shaped, and the peak-to-background ratio was favorable (in comparison with acetate buffer), the mentioned electrolyte was chosen as optimal for further measurements. 

#### 2.4.2. Concentration of Supporting Electrolyte

Consequently, the next tested parameter was the optimal concentration of hydrochloric acid used as a supporting electrolyte. The conditions of the experiment were analogous to the ones described above. Hydrochloric acid concentration values were tested in a range from 0.01 mol L^−1^ to 0.25 mol L^−1^. The obtained results revealed that the concentration of the electrolyte has no influence on the QY peak current, which was about 1.80 μA. However, the value of this parameter influenced QY peak potential—when the hydrochloric acid concentration was increased; the peak potential shifted toward less negative values (−646 mV for 0.01 mol L^−1^ and −593 mV for 0.25 mol L^−1^). After analysis of the obtained results (peak shape, peak-to-background ratio, peak position), a hydrochloric acid concentration equal to 0.05 mol L^−1^ was chosen as optimal. 

### 2.5. Preconcentration Potential and Time Optimization

#### 2.5.1. Preconcentration Potential

The influence of the preconcentration potential on the QY signal was investigated. During the experiment, the supporting electrolyte consisted of 0.05 mol L^−1^ hydrochloric acid, the preconcentration time was equal to 20 s, and the QY concentration was 0.2 µmol L^−1^. Values of preconcentration potential varied in the range from 70 mV to −400 mV. Based on the obtained results, it was concluded that the value of the preconcentration potential did not affect the maximum current of the QY peak—its value was equal to 1.80 µA. There was also no influence of the tested parameter on the value of the QY peak potential, which was equal to approximately −608 mV. Based on the presented results, the 50 mV preconcentration potential was chosen as optimal for further measurements. 

#### 2.5.2. Preconcentration Time

**Figure 5:**
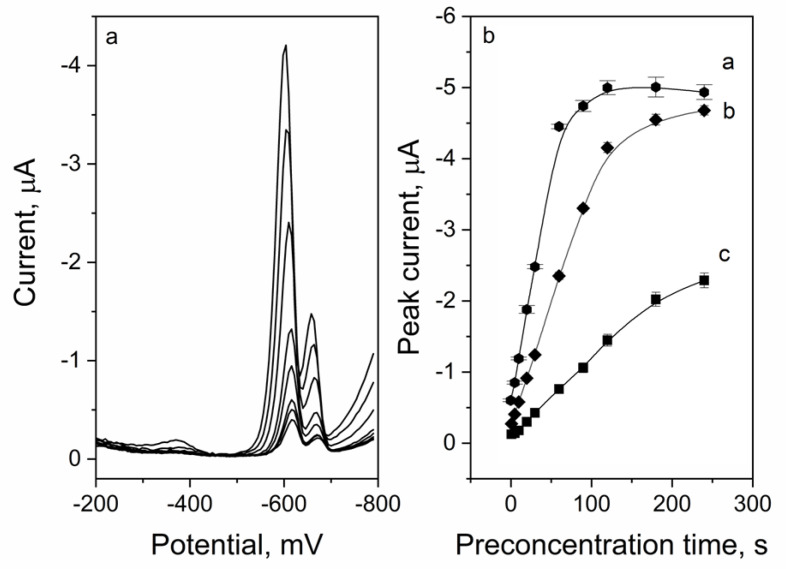
DPV preconcentration plots of quinoline yellow registered for QY concentration (a) 2 × 10^−7^ mol L^−1^, (b) 1 × 10^−7^ mol L^−1^, and (c) 2.5 × 10^−8^ mol L^−1^ in 0.05 mol L^−1^ HCl (**b**) and corresponding voltammograms for a concentration of 1 × 10^−7^ mol L^−1^ and preconcentration time from 0 to 240 s (**a**).

In stripping techniques, elongation of the preconcentration time often results in a higher sensitivity of measurements. Therefore, in the next performed experiment, the influence of preconcentration time on the QY signal was investigated. The conditions of the experiment were analogous to those described above; the preconcentration potential was equal to 50 mV. The preconcentration time was varied in the range of 0–240 s, and the results obtained are presented in Figure 5. As it can be observed, the longer the preconcentration time, the higher the peak current of QY registered (e.g., for a QY concentration of 1 × 10^−7^ mol L^−1^: for preconcentration of 0 s I_p_ = 0.60 μA, for 240 s I_p_ = 4.94 μA). The most effective increase in the signal was observed up to approximately 90 s of preconcentration time—after exceeding this value; the peak current started to stabilize. A value of preconcentration potential slightly influences the value of the QY peak potential—when the time is longer, the peak starts to shift toward less negative potentials. 

### 2.6. Interference Study

The interference study is an important step in the context of real sample measurements using a new analytical method. The study of the influence of potential matrix components allows for the development of a sample preparation strategy. Experimental conditions were as follows: supporting electrolyte consisted of 0.05 mol L^−1^ hydrochloric acid, *E_acc_* = 50 mV, *t_acc_* = 20 s, and concentration of QY equal to 0.2 µmol L^−1^. Among the possible interferents, the influence of a group of metals such as Cu(II), Mn(II), Fe(III), Zn(II), Pb(II) (2 µmol L^−1^ added), Ca(II), Mg(II) (20 µmol L^−1^ added) was tested on the QY signal. In addition, a few organic substances were investigated, such as citric acid, glucose, saccharose (2 µmol L^−1^ added), and one nonionic surfactant—Triton X-100 (2 ppm added). The experiment revealed that the presence of Cu(II) ions at a concentration 2 µmol L^−1^ (interferent to analyte ratio 10:1) caused a 25% decrease in the signal. Triton X-100 also caused deterioration of measurement conditions—1 ppm resulted in a 15% drop in the signal, while 2 ppm decreased the peak current by 85%. Other tested substances had no significant influence on the QY signal. 

### 2.7. Analytical Performance

**Figure 6:**
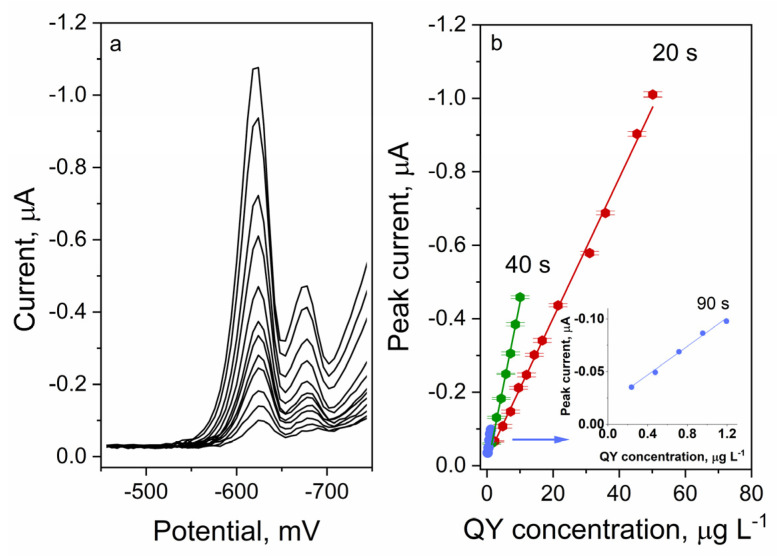
DPV calibration curves of quinoline yellow registered for preconcentration times of 20 s, 40 s, and 90 s in 0.05 mol L^−1^ HCl (**b**) and (**a**) corresponding voltammograms for preconcentration time of 20 s and concentration range from 2.4 to 50.1 µmol L^−1^.

The linear range and detection limits of quinoline yellow were investigated using the DPV voltammetry technique under optimized conditions (Figure 6). The detection limit obtained for a shorter preconcentration time of 20 s was equal to 3.72 nmol L^−1^ with linearity up to 105 nmol L^−1^. Lengthening the preconcentration time to a value of 90 s results in obtaining a lower detection limit with the value of 0.48 nmol L^−1^, which is a very good result compared to other voltammetric techniques used for high-sensitive QY determination. The obtained analytical data compared to previously reported results are presented in Table 1. 

**Figure 7:**
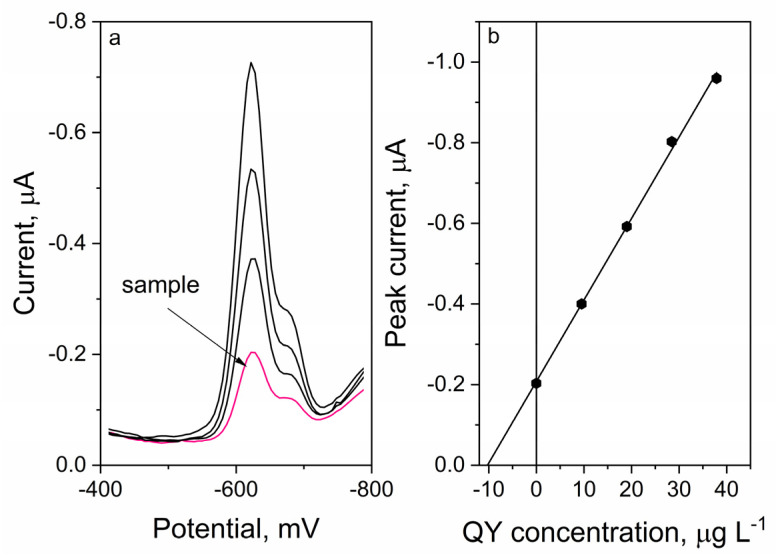
DPV voltammograms of quinolone yellow measured in Drink 2 sample (Drink 2 curve marked with pink color) (**a**) with corresponding calibration curve (**b**).

Once all experimental variables were optimized, the performance of the Hg(Ag)FE electrode was evaluated for the determination of QY in beverages, powder, and throat lozenges available in Poland. Samples were purchased from local markets and pharmacies, and the procedures of sample preparation are described in Section 3.3. After adding 50 µL of the previously prepared sample to the supporting electrolyte consisting of 50 µL 10 mol L^−1^ HCl and 9.9 mL of double distilled water, the QY DPV curves were registered. The registered voltammograms with the according calibration plot of the QY determination in the drink samples are presented in Figure 7. The results of the performed measurements using the standard addition method with recovery parameters for all tested samples are presented in Table 2. Each sample curve was registered three times, and the reproducibility of the measurements measured and expressed as RSD value was approximately 2% (QY concentration 0.2 µmol L^−1^, n = 5), confirming the excellent precision of the proposed method. The measured recovery was very good, ranging from 96 to 106%. In order to verify the accuracy of the developed voltammetric method, UV-VIS spectrophotometry was used as a reference method. Samples measurements were conducted using the calibration method with a series of QY standard solutions. The obtained results (Table 2) were consistent with the ones obtained using voltammetry. 

## 3. Materials and Methods

### 3.1. Apparatus

All voltammetric measurements were conducted using an electrochemical analyzer M161 and electrode stand M164—mtm-anko (Krakow, Poland). A typical three-electrode system consisting of silver–silver chloride (3 mol L^−1^ KCl) (reference electrode), platinum wire (auxiliary electrode), and the renewable amalgam film electrode Hg(Ag)FE (working electrode) was used. The working electrode was stable during all performed measurements and did not require regeneration. The detailed process of its preparation and construction was presented in Ref. [22]. Voltammetric data were registered and processed using EaQt software (Krakow, Poland, https://github.com/efce/EAQt/blob/master/eaqtparampotentialprogram.cpp, accessed on 13 July 2023). A solution in an electrochemical quartz cell was stirred using a magnetic stirrer with a speed of ~500 rpm. All pH measurements were carried out using a laboratory pH meter. For spectrophotometric studies, a UV-VIS spectrophotometer with a double beam equipped with deuterium and halogen lamps (JASCO V-630, Tokyo, Japan) was used. Measurements of absorption spectra (in the range 240–500 nm) and absorbance (425 nm) were conducted in quartz cuvettes (optical path length 10 mm). The scan speed was equal to 4000 nm min^−1^.

### 3.2. Chemicals and Glassware

All chemicals used were of analytical grade and were utilized without further purification. Quinoline yellow was purchased from Sigma Aldrich (Sigma Aldrich, St. Louis, MO, USA). A stock solution used for measurements with a concentration of 0.01 mol L^−1^ was prepared by dilution in double distilled water, while lower concentrations were prepared daily. Hydrochloric acid was purchased from Merck, (Darmstadt, Germany), and Triton X-100 from Windsor Laboratories Ltd., UK (Kingston, Jamaica). All aqueous solutions were prepared using double distilled water. Borosilicate glass utilized during experiments was cleaned using HNO_3_ and double distilled water.

### 3.3. Samples Preparation

#### 3.3.1. Liquid Samples

QY determination was carried out in two isotonic drinks with QY in their composition, labeled as Drink 1 and Drink 2, and one fizzy drink without QY in its composition, labeled as Drink 3. Before the experiment, all samples were simply diluted in double distilled water and measured without further preparation.

#### 3.3.2. Powder

The investigated pre-workout supplement (lemon flavor) is a nutridrink in the form of powder (should be dissolved in water before use), mainly aimed at people who are physically active. It increases the amount of protein in the diet, which helps to build muscle mass. For quantitative measurement of QY, 1 g of sample was dissolved in 5 mL of double distilled water and filtrated using a cellulose syringe filter (pore size 0.2 µm). The obtained filtrate was transferred into a 10 mL volumetric flask and filled with double distilled water.

#### 3.3.3. Tablet

A popular throat lozenge with honey and lemon flavor was used as a sample in the form of a tablet. For quantitative QY measurements, one tablet was placed in a beaker with 25 mL of double distilled water and sonicated until complete dissolution (approx. 20 min). The obtained solution was transferred into the 50 mL volumetric flask and filled up with double distilled water.

### 3.4. Measurement Procedure

The DPV method and standard addition method were used for all quantitative measurements. Before the experiment, an electrochemical cell was filled with 10 mL of supporting electrolyte consisting of 0.05 mol L^−1^ hydrochloric acid. Values of *E_acc_* and *t_acc_* were equal to 50 mV and 20 s, respectively. Before measurement, the electrolyte was de-aerated using continuous argon flow (5N purity) for approx. 5 min. Hg(Ag)FE with the size of a surface area of 9.8 mm^2^ was used as the working electrode. The surface of the electrode was refreshed before each signal registration. Each measurement was performed according to the following procedure:

- Cleaning of the working electrode’s surface: −1000 mV
- Preconcentration of QY: *t_acc_* = 20 s, *E_acc_* = 50 mV
- Rest period: 3 s
- Voltammogram registration in the range from 50 mV to −850 mV

The remaining parameters of the DPV technique for QY determination were as follow: sampling and waiting time *t_s_ = t_w_* = 10 ms, pulse step *Es* = 6 mV, pulse amplitude Δ*E* = 50 mV.

### 3.5. Data Analysis

During all voltammetric experiments, each measurement was repeated at least three times to gather data for statistical analysis. Data analysis started with the correction of the baseline, signal smoothing using the Savitzky–Golay filter, and data averaging. Average values and standard deviations of peak current and peak potential were determined. Data obtained were used for the preparation of graphs, while the standard deviation was presented in the form of error bars.

## 4. Conclusions

In this work, a mercury film electrode with a renewable surface (Hg(Ag)FE) was successfully applied for high-sensitivity quinoline yellow determination for the first time. Due to its large working area, it was possible to reach a low detection limit, equal to 0.48 nmol L^−1^, which is a very good result compared to other previously reported voltammetric measurements. Simplicity, high repeatability, sensitivity of the measurements, and short time of sample analysis are the main advantages of the proposed method. The QY analysis in the real samples validated the potential utility of the used sensor for routine quality control analysis, which was also proven by the satisfactory values of the recovery parameter (96–107%). The accuracy of the proposed sensor was confirmed by using UV-VIS spectrophotometry as the reference method. 

## Figures and Tables

**Table 1:** Comparison of quinoline yellow determination methods.

Method	Detection Limit	Source
HPLC	92.2 pmol L^−1^	[10]
RP-HPLC	0.16 µmol L^−1^	[9]
Spectrophotometry	0.34 µmol L^−1^	[4]
Spectrophotometry	34.4 µmol L^−1^	[5]
Spectrophotometry	4.4 nmol L^−1^	[8]
Spectrophotometry	28.1 nmol L^−1^	[7]
Fluorescence	0.11 µmol L^−1^	[20]
Potentiometry	40 µmol L^−1^	[21]
DPV (GC)	0.25 mmol L^−1^	[14]
CV (MWCNT/ITO)	4 nmol L^−1^	[19]
DPV (PDDA-RGO)	2 nmol L^−1^	[12]
SDLSV (MnO_2_/GR/GC)	0.04 µmol L^−1^	[15]
DPV (PVP/CPE)	27 nmol L^−1^	[16]
DPV (MWCNT/GC)	1.0 µmol L^−1^	[17]
DPV (ZnO-Pd/GC)	4 nmol L^−1^	[18]
DPV (WS_2_/DNPs/GC)	3.4 µmol L^−1^	[11]
SWV (PPy–SWCNT/GC)	0.08 µmol L^−1^	[13]
DPV (Hg(Ag)FE)	0.48 nmol L^−1^	This work

HPLC—High Performance Liquid Chromatography; RP-HPLC—Reversed Phase High Performance Liquid Chromatography; DPV (GC)—Differential Pulse Voltammetry with Glassy Carbon Electrode; CV (MWCNT/ITO)—Cyclic Voltammetry with Glassy Carbon electrode modified with Multiwalled Carbon Nanotubes with Indium Tin Oxide; DPV (PDDA-RGO)—Differential Pulse Voltammetry Glassy Carbon electrode modified with Polydiallyldimethylammonium Chloride functionalized reduced Graphene Oxide nanocomposite; SDLSV (MnO_2_/GR/GC)—Second Derivative Linear Sweep Voltammetry with Glassy Carbon Electrode modified with Manganese dioxide and Graphene; DPV (PVP/CPE)—Differential Pulse Voltammetry with Polyvinylpyrrolidone modified Carbon Paste Electrode; DPV (MWCNT/GC)—Differential Pulse Voltammetry with Glassy Carbon Electrode modified with Multiwalled Carbon Nanotubes; DPV (ZnO-Pd/GC)—Differential Pulse Voltammetry with Glassy Carbon Electrode modified with Zinc Oxide—Palladium Nanocomposite; DPV (WS_2_/DNPs/GC)—Differential Pulse Voltammetry with Glassy Carbon Electrode modified with 2D Tungsten Disulphide and Diamond Nanoparticles; SWV (PPy–SWCNT/GC)—Square Wave Voltammetry with Glassy Carbon Electrode modified with Polypyrrole and Single-walled Carbon Nanotubes; DPV (Hg(Ag)FE)—Differential Pulse Voltammetry with the renewable amalgam film electrode.

**Table 2:** Results of QY determination in beverages, powder, and throat lozenges samples.

	Voltammetry		Spectrophotometry
Sample	QY Added, mg L^−1^	QY Found ± s, mg L^−1^, (Recovery, %)	QY Found, mg L^−1^
Drink 1	0	21 ± 2	22 ± 2
	19	40 ± 4 (96)	-
	38	59 ± 2 (100)	-
	57	78 ± 7 (106)	-
Drink 2	0	18 ± 3	20 ± 2
	19	37 ± 1 (99)	-
	38	56 ± 1 (99)	-
	57	75 ± 1 (96)	-
Drink 3	0	ND	ND
	19	19 ± 1 (102)	-
	38	40 ± 1 (105)	-
	57	58 ± 1 (101)	-
**Sample**	**Added, mg kg^−1^**	**Found ± s, mg kg^−1^**	**Found ± s, mg kg^−1^**
Pre-workout supplement (powder)	0	1.15 ± 0.06	1.17 ± 0.03
	0.95	0.95 ± 0.02 (100)	-
	1.9	1.9 ± 0.01 (101)	-
	2.85	2.86 ± 0.01 (102)	-
Throat lozenges (tablet)	0	0.30 ± 0.02	0.27 ± 0.04
	0.95	0.92 ± 0.03 (97)	-
	1.9	1.78 ± 0.07 (95)	-
	2.85	2.81 ± 0.04 (98)	-

ND—not detected.

## Data Availability

The data presented in this study are available on request from the corresponding author.
